# Testicular versus ejaculated sperm should be used for intracytoplasmic sperm injection (ICSI) in cases of infertility associated with sperm DNA fragmentation | *Opinion: No*

**DOI:** 10.1590/S1677-5538.IBJU.2018.04.04

**Published:** 2018

**Authors:** Mark Sigman

**Affiliations:** 1 Department of Urology Brown University and The Miriam Hospitals, RI 02906, EUA

**Keywords:** Semen, Infertility, Male, Sperm Injections, Intracytoplasmic, Sperm DNA Fragmenttion, Testicular Sperm

The argument for the use of testicular sperm instead of ejaculated sperm for infertility due to sperm DNA fragmentation (SDF) relies on several assumptions. When each assumption is examined, it becomes clear that the assumptions are either unproven, due to insufficient data, or just plain wrong. These assumptions are: 1) sperm DNA fragmentation assays are good diagnostic tests; 2) IVF/ICSI failed because of elevated SDF; and 3) testicular sperm will result in pregnancy or live birth when ejaculated sperm will not. It has been demonstrated that when comparing populations, SDF is greater in infertile than in fertile populations. In addition, SDF is negatively associated with pregnancy rates by IVF/ICSI with an odds ratio of 1.68 (1). However, these population associations are insufficient to rely of SDF assays to direct patient management. There are a multitude of SDF assays, some such as TUNEL, directly measure the presence of sperm DNA fragmentation, while others such as SCSA, alkaline COMET, and Sperm Chromatin Dispersion (SCD) only indirectly measure fragmentation. There remains poor standardization of techniques and even variable protocols of the same assays between different laboratories. These problems have lead the American Society of Reproductive Medicine Practice Committee report to state that “existing data relating to relationship between DNA fragmentation and reproductive outcomes too limited to routinely use” Their most favorable conclusion is that the effect of SDF on IUI, IVF, and ICSI may be clinically informative - hardly an overwhelming endorsement (2). While odds ratios are useful for describing associations in populations, they are not proper statistical metrics by which diagnostic tests are judged. When the results of individual patient's SDF assays are used to direct those individual's therapy, the assays are being used as diagnostic tests. The metrics by which diagnostic tests are evaluated are sensitivity, specificity, and the area under the Receiver Operating Characteristic (ROC) curves. The relationship between SDF and ART outcomes have been evaluated by at least 7 prior meta-analyses - all of which used odds ratios or relative risk ratios. Overall the results have been inconclusive. Li et al. reported that SDF was associated with IVF pregnancy rates but not with ICSI outcomes (3). Collins reported that sperm DNA damage predicts ART outcome, but the results of testing would not necessarily affect the decision to proceed with ART because the effects were likely clinically insignificant (4). Most recently, Simon determined that the association depends on the type of assay used. There was no association of SCSA and ART outcomes while there was a negative relationship between SDF and IVF and/ or ICSI outcomes. When proper ROC analysis is performed, the assays perform either mediocre or no better than flipping a coin. For example, the TUNEL has an AUC of 0.71, a sensitivity of 0.84 but a specificity of 0.24. This means that there is there is a high false positive rate - patients with elevated SDF still become pregnant. This makes it inaccurate to use the results to inform patients that they will not achieve pregnancy by ART using ejaculated sperm if their SDF by TUNEL is elevated. In comparison, the SCSA and SCD had AUC's of 0.49 - no better than using a coin flip to determine the result and make clinical recommendations (5). It is clear that recommending testicular sperm retrieval (TESE) for couples with high SDF is based on a test that needs improvement in testing characteristics, validation in clearly defined populations, and standardization of protocols and thresholds.

The use of testicular sperm instead of ejaculated sperm assumes that testicular sperm is of better quality. In comparing testicular to ejaculated sperm in the same patients, testicular sperm has been found to have lower SDF, however, sperm aneuploidy was increased in the testicular sperm - certainly a worrisome finding (6). To justify TESE instead of ejaculated sperm, we must be assured that testicular sperm are healthier sperm, not just sperm with lower SDF values. Regardless of the biology of the sperm, testicular sperm should yield better pregnancy rates than ejaculated sperm. A recent meta-analysis found no statistically significant improvement in ICSI pregnancy rates in cryptozoospermic men when comparing testicular sperm to ejaculated sperm (7).

Many studies arguing for TESE report pregnancy after TESE/ICSI cycles in couples that have failed prior ejaculated sperm ICSI cycles. The assumption is that the pregnancies in subsequent ICSI cycles were due to the use of testicular sperm, not due to just repeating the IVF/ICSI cycle. These studies ignore the fact that pregnancy and live birth rates are substantial with further ejaculated sperm IVF/ICSI cycles. Luke et al. utilizing SART data calculated estimated optimal cumulative live birth rates of approximately 30% after 1 cycle growing to over 60% by the 3rd cycle with further increases with additional cycles (8). Simply put, couples that fail an IVF/ICSI cycle, may become pregnant by just pursuing additional ejaculated sperm cycles without resorting to TESE. The argument for utilizing TESE sperm for couples that have failed IVF/ ICSI relies on studies reporting better pregnancy rates with this approach. It is quite informative to examine the studies and the study designs supporting this approach. There are three types of study designs that can evaluate ejaculated vs. testicular sperm. Case series are the most frequently published manuscripts. These papers report pregnancy rates in couples that utilized testicular sperm after they failed ejaculated sperm cycles. It is obvious that since the initial ejaculated sperm cycles failed to achieve pregnancy that pregnancy rates in subsequent cycles will be better if they occur at all. All couples who achieved pregnancy with ejaculated sperm are excluded from these reports. Thus, this study design cannot demonstrate that the cause of subsequent successful cycles was due to the source of the sperm or just from repeating the cycles. A second study design reported are cohort studies in which couples that choose TESE are compared to those that refused TESE. While this study design is superior to case study designs, the one study utilizing this approach proceeded to TESE based on SDF results without having the couples under an initial ejaculated sperm ICSI cycle. Thus, we don't know that the couples in the TESE arm would have failed ICSI with ejaculated sperm. In addition, since patients chose which treatment they wanted, the door is open to biasing factors that are not accounted for (9). The ideal study design is a randomized controlled trial of couples that failed IVF/ICSI, including groups with normal and elevated SDF in both arms that are randomized to either ejaculates sperm ICSI or testicular sperm ICSI. It is important to include subgroups of normal SDF since the argument for TESE in these cases is that the ejaculated sperm have high SDF and the testicular sperm have lower SDF and that outcomes from testicular sperm are limited to those with high SDF. Without examining couples with low ejaculated sperm SDF, any demonstrated improvement in pregnancy rates may be due to just using testicular sperm - not due to lower SDF in testicular sperm. Review of these randomized studies is easy because there are none, not a single one. All reports are either case series or observational cohort designs, and only include couples with high SDF. Up through the end of 2017, there are five reports (four published articles, and one abstract) comparing pregnancy rates from testicular and ejaculated sperm in couples with elevated SDF. All report better pregnancy or live birth rates from testicular sperm. While a superficial review suggests the superiority of testicular sperm, an evaluation of the study designs suggests otherwise. Three publications are case series that compared subsequent testicular sperm cycles to prior failed cycles in the same patients (10-12). As discussed, results can only be better in subsequent cycles, and as demonstrated by the SART data analysis, repeated cycles are often successful. Two cohort studies compared two different patient groups, all of whom had elevated SDF - ejaculated sperm couples and testicular sperm couples. In one study, couples went to TESE based on elevated SDF without having had any prior ICSI cycles (9). The second only included couples that failed prior IVF/ICSI cycles (13). Since these two studies utilized completely different study designs addressing different questions, they cannot but combined to address this issue.

Examining the characteristics of subjects included in the various studies is critical to allow generalizations that we may make to apply the findings to our own patients. Populations consisted of only patients with sperm densities of less than 5 million sperm per mL (11), sperm densities of 5 – 15 million/ mL (9), normal sperm densities (13), while some contained subjects with wide ranges of semen parameters (10, 12). These differences between subject populations makes it difficult to compare studies. Further difficulties arise when examining what level of sperm DNA fragmentation was considered elevated in these studies. Of the studies utilizing TUNEL assays, thresholds were >30% (13), >15% (10), and >7% (11). Of those that used the SCD, thresholds were 30% but the assay techniques were different (9, 12). Therefore, some studies classified patients as abnormal while those same patients would have been classified as normal by other investigators. Outcomes that may be examined include fertilization rates, pregnancy rates, miscarriage rates, and live birth rates. Most studies reported no difference in fertilization rates between TESE and ejaculated groups while one reported worse rates in the TESE group (9). While four of the studies reported better pregnancy rates and one reported no change, because of the study design limitations, the cause of the better pregnancy rates cannot be attributed to the source of sperm. Similarly, live birth rates were reported as better with testicular sperm (one study did not report live birth rates), the study designs limit conclusions about the cause of the differing rates. Most recently, a study presented in May 2018 examined couples with elevated SDF that failed an ejaculated sperm ICSI cycle who subsequently choose either TESE or a second ejaculated sperm ICSI cycle. The authors found no statistical difference in pregnancy or live birth rates when comparing the testicular sperm to the ejaculated sperm group (14). Of interest, in the two studies reporting miscarriage rates, both found lower rates in the testicular groups (9, 10). This raises the possibility that better live birth rates may be due to lower miscarriage rates from testicular sperm and that what we should study is not infertility couples, but recurrent miscarriage couples.

This methodical analysis of the published data demonstrates that SDF tests fail as diagnostic tests to direct therapy. The assays need standardization and validation. In addition, the published studies do not utilize one of the most commonly ordered and commercially available assays, the SCSA. Therefore, clinicians should not extrapolate their patient's SCSA results to determine whether to use TESE for ICSI cycles. Elevated SDF has not been shown to be the cause of failed ART cycles due to flaws in study designs and the lack of data on low SDF couples in these trials. Testicular sperm has not been shown to result in better outcomes than ejaculated sperm since the level of evidence is poor - ranging from 2b (cohort studies) to 4 (case series). The included subjects are not comparable with greatly variable sperm densities in different studies. The practice of testicular sperm retrieval for couples with elevated SDF and failed IVF/ICSI should be considered experimental - randomized controlled trials are greatly needed. In addition, the potential genetic and epigenetic risks of testicular sperm should not be ignored. Finally, it is important to remember that we physicians have taken an oath to do no harm.
